# Subsequent Proceedings before the Lord Chancellor and the Lords Justices of Appeal

**Published:** 1852-04-01

**Authors:** 


### SUBSEQUENT PROCEEDINGS

BEFORE THE

### LORD CHANCELLOR AND THE LORDS JUSTICES OF APPEAL.

---

Mrs. Cumming petitioned the court to traverse the inquisition: the petition was opposed by the promoters of the Commission. Lengthened arguments ensued upon the question, whether the issuing of the traverse was a matter of right. On the 27th of March, 1852, the case was decided, after the Lord Chancellor had seen the alleged lunatic. The following is an extract from the *Times* report of his lordship's judgment:—

"The Lord Chancellor held, that the petitioner had a right to traverse the inquisition as of course. Lord Eldon had stated the duty of the court to be, to satisfy itself that the application was *bonâ fide*, and that the lunatic was desirous that it should issue, and in that event to allow the traverse to issue . . . . Following that rule, he (the Lord Chancellor), without throwing the duty upon the Lords Justices, had that morning had a lengthened interview with the lunatic, and, after reasoning with her, and representing to her the great expense that would be incurred by a further inquiry, he must say, that upon that point she was as rational and sane as any person that he had ever conversed with. Without, therefore, expressing any opinion as to whether she laboured under any particular delusions, he (the Lord Chancellor) must say that she appeared to him perfectly competent to judge whether she really wished the traverse should be allowed or not. When before him, she expressed herself mildly, without any passion whatever, that she was desirous the traverse should issue, although informed of the probable extent to which her property would thereby be imperilled. She declared she was content to make any sacrifice, and to submit to any terms, by which she might obtain 'liberty of action.' She satisfied his mind that the present application was made of her own free will, and expressed a hope that he would grant it, if he thought proper to do so. Under those circumstances, the Court was bound to say that the traverse must go."

Lords Justices Knight Bruce and Cranworth severally concurred with the judgment of the Lord Chancellor.

